# Divergent effects of RIP1 or RIP3 blockade in murine models of acute liver injury

**DOI:** 10.1038/cddis.2015.126

**Published:** 2015-05-07

**Authors:** M Deutsch, C S Graffeo, R Rokosh, M Pansari, A Ochi, E M Levie, E Van Heerden, D M Tippens, S Greco, R Barilla, L Tomkötter, C P Zambirinis, N Avanzi, R Gulati, H L Pachter, A Torres-Hernandez, A Eisenthal, D Daley, G Miller

**Affiliations:** 1 S Arthur Localio Laboratory, Department of Surgery, New York University School of Medicine, 550 First Avenue, New York, NY, USA; 2 Department of Cell Biology, New York University School of Medicine, 550 First Avenue, New York, NY, USA

## Abstract

Necroptosis is a recently described Caspase 8-independent method of cell death that denotes organized cellular necrosis. The roles of RIP1 and RIP3 in mediating hepatocyte death from acute liver injury are incompletely defined. Effects of necroptosis blockade were studied by separately targeting RIP1 and RIP3 in diverse murine models of acute liver injury. Blockade of necroptosis had disparate effects on disease outcome depending on the precise etiology of liver injury and component of the necrosome targeted. In ConA-induced autoimmune hepatitis, RIP3 deletion was protective, whereas RIP1 inhibition exacerbated disease, accelerated animal death, and was associated with increased hepatocyte apoptosis. Conversely, in acetaminophen-mediated liver injury, blockade of either RIP1 or RIP3 was protective and was associated with lower NLRP3 inflammasome activation. Our work highlights the fact that diverse modes of acute liver injury have differing requirements for RIP1 and RIP3; moreover, within a single injury model, RIP1 and RIP3 blockade can have diametrically opposite effects on tissue damage, suggesting that interference with distinct components of the necrosome must be considered separately.

The etiologies of acute liver injury are diverse and its overall public health burden is considerable. Liver injury from acetaminophen (APAP) overdose is the most common cause of death from over-the-counter drugs and is the leading cause of acute liver failure in the developed world.^1, 2, 3^ Hepatic dysfunction from autoimmune hepatitis has a prevalence of 10–20/100 000.^4, 5^ Other etiologies of acute liver failure include idiosyncratic reaction to medications such as tetracycline, severe viral or alcoholic hepatitis, acute fatty liver of pregnancy, and idiopathic causes. Clinical complications resulting from liver failure include hepatic encephalopathy, impaired protein synthesis, and coagulopathies. Moreover, there are no effective means to reverse liver failure once advanced disease sets in – regardless of etiology – and transplantation frequently remains the only option for survival.^6^

Concanavalin-A (ConA) is a lectin derived from the jack-bean plant with a unique ability to induce hepatitis in a well-described murine model of acute hepatic injury. ConA stimulates mouse CD4^+^ T-cell subsets to mediate insult to hepatocytes. The resulting cytokine release can further lead to recruitment and activation of innate inflammatory mediators, which perpetuate an insidious cycle of inflammation and hepatocellular injury.^7, 8, 9^

APAP is a widely used analgesic and antipyretic. Although usually considered safe at therapeutic doses, at higher doses APAP causes acute liver failure characterized by centrilobular hepatic necrosis.^1, 10^ At therapeutic doses, >90% of APAP is metabolized by glucuronidation and sulphation and its metabolites are excreted via the renal system. Of the remaining APAP, roughly 2% is excreted intact in the urine, and approximately 8% is metabolized by the cytochrome P450 system to *N*-acetyl-p-benzo-quinone imine (NAPQI), which is highly reactive.^11, 12^ Hepatic glutathione (GSH) then induces the formation of a safely excretable APAP-protein adduct. However, at toxic doses of APAP, GSH becomes depleted and NAPQI is able to exert harmful effects by forming covalent bonds with mitochondrial proteins, inhibiting the Ca^2+^-Mg^2+^-ATPase and inducing mitochondrial dysfunction.^1, 2^ This disturbance leads to a decrease in ATP synthesis, disruption of cellular membrane, and eventually hepatocyte death.^13^

Although GSH depletion and the resulting toxic metabolites are prerequisites for APAP hepatotoxicity, there is evidence that the severity of liver injury may depend on subsequent participation of innate immunity.^10, 14, 15, 16^ In particular, APAP-induced injury has been reported to be contingent on activation of the NLRP3 inflammasome via DAMPs released from injured hepatocytes. Inflammasome activation cleaves Caspase 1 inducing IL-1*β* release and galvanizing intrahepatic neutrophils and inflammatory monocytes, which exacerbate injury.^17^ However, alternate studies using transgenic mice suggest that NLRP3 inflammasome is largely dispensable for APAP toxicity.^18^ Thus the role of inflammasome activation in APAP toxicity is controversial and may be dependent on the precise experimental conditions or strain of mice employed.

Apoptosis and necrosis are classically understood processes of cell death that denote either organized Caspase 8-dependent programmed cell death or non-programmed disorganized death, respectively. In contrast to necrosis, which leads to the release of DAMPs and sustains inflammation, apoptosis produces cell fragments called apoptotic bodies, which phagocytic cells are able to engulf before the contents of the cell can spill out onto the surrounding space and activate innate immunity. ‘Necroptosis' is a recently described Caspase 8-independent method of cell death that denotes organized cellular necrosis. Necroptosis requires the co-activation of RIP1 and RIP3 kinases. Both *in vitro* and *in vivo* investigations have suggested that APAP can induce cellular demise via necrosis or Caspase 8-dependent apoptosis, which is determined, in part, by ATP availability from glycolysis.^19^ Zhang *et al.*^20^ recently confirmed that RIP1 is necessary in APAP-induced necroptosis. Similarly, Takemoto *et al.*^21^ showed that RIP1 inhibition protects against reactive oxygen species (ROS)-induced hepatotoxicity in APAP-induced acute liver injury. Further, a recent report suggested that selective inhibition of RIP3 using the anticancer drug Dabrafenib alleviates APAP injury.^22^

In the ConA model of acute liver injury, experiments using apoptosis-resistant mice expressing mutant FADD revealed that ConA alone induced primarily necrotic cell death, whereas ConA combined with d-galactosamine induced apoptosis and necrotic cell death.^23^ Zhou *et al.*^24^ reported that Necrostatin-1 (Nec-1) prevents autoimmune hepatitis in mice via RIP1- and autophagy-related pathways. Another recent report investigated the role of RIP1, RIP3, and PARP-1 in murine autoimmune hepatitis. This study found that in cases where death of mouse hepatocytes is dependent on TRAIL and NKT cells, PARP-1 activity was positively correlated with liver injury and hepatitis was prevented both by RIP1 or PARP-1 inhibitors.^25^ Our goal in the current study was to investigate, in parallel, the effects of RIP1 and RIP3 blockade in diverse models of acute liver injury. Our work suggests that modulating necroptosis may have divergent effects, depending on the etiology of hepatic injury and the specific component of the necrosome being targeted.

## Results

### Evidence of elevated RIP1 and RIP3 expression in acute liver injury

**Figure 1:**
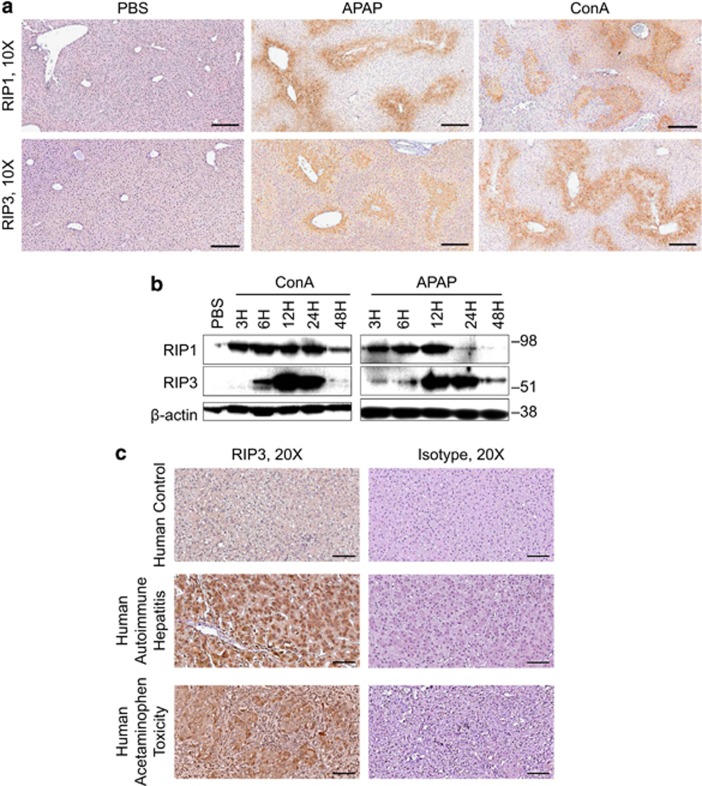
Necrosome activation in acute liver injury in mice and humans. (**a**) Expression of RIP1 and RIP3 at 12 h was tested by immunohistochemistry on paraffin-embedded liver sections from control, ConA- (20 *μ*g/g), and APAP (500 *μ*g/g)-treated mice (scale bar=200 *μ*m). (**b**) Expression of RIP1 and RIP3 was tested by western blotting in the control liver or at various time points after administration of ConA or APAP. *β*-Actin was used as a loading control. (**c**) Paraffin-embedded human liver sections from a patient with normal liver or from patients who underwent liver transplant for autoimmune hepatitis or acute APAP toxicity were tested for expression of RIP3 by IHC compared with isotype control (scale bar=100 *μ*m)

To investigate the expression of components of the necrosome in acute liver injury, we tested RIP1 and RIP3 expression in the normal liver and the liver of APAP- or ConA-treated mice. We found the absence of RIP1 and RIP3 expression in normal hepatocytes but extensive expression in the liver from APAP- or ConA-treated mice on immunohistochemical analysis and western blotting (Figures 1a and b). Notably, FADD was modestly upregulated after APAP injury, and Caspase 8 was expressed at higher levels in the acutely injured liver, whereas c-FLIP was not markedly changed (Supplementary Figure S1). Similarly, our analysis of human liver tissues revealed elevated expression of RIP3 in the liver of patients with hepatic failure from autoimmune hepatitis or severe APAP toxicity, but expression was absent in the normal human liver (Figure 1c).

### RIP3 deletion delays hepatic injury in ConA hepatitis

**Figure 2:**
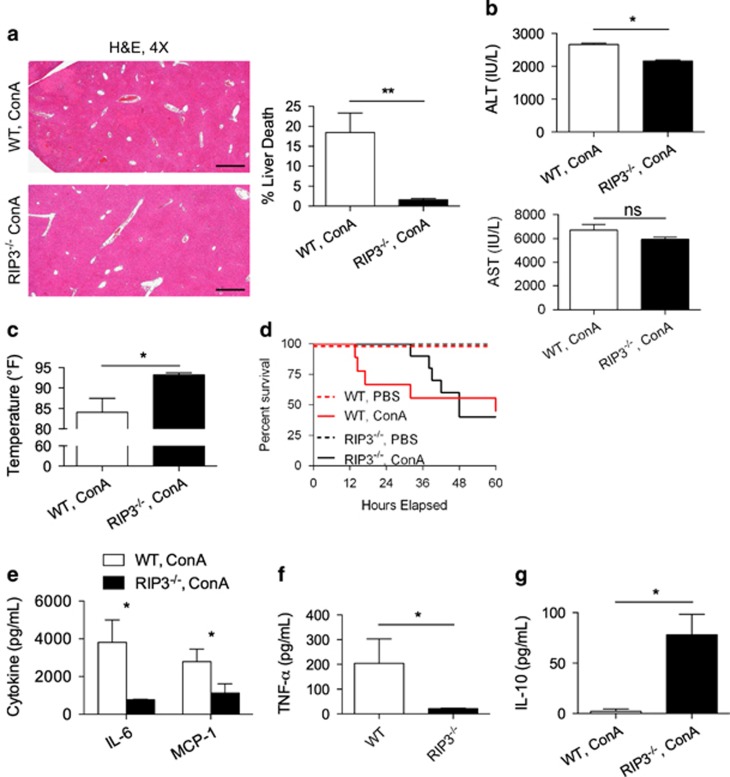
RIP3 deletion protects against ConA hepatitis. (**a**–**c**) WT and RIP3^−/−^ mice were treated with ConA (20 μg/g). (**a**) Hematoxylin–eosin (H&E)-stained sections of the liver harvested 12 h after injury are shown. The fraction of non-viable liver area was quantified (*n*=10/group) (scale bar=500 *μ*m). (**b**) Serum ALT, aspartate aminotransferase (AST), and (**c**) core body temperature were measured at 12 h. (**d**) WT and RIP3^−/−^ mice were administered a lethal dose of ConA (40 *μ*g/g). Kaplan–Meier survival analysis was performed (*n*=9–10/group; *P*=NS). (**e**–**g**) WT and RIP3^−/−^ mice were treated with ConA (20 μg/g). (**e**) Serum levels of interleukin (IL)-6 and MCP, (**f**) hepatic NPC expression of TNF-α, and (**g**) serum levels of IL-10 were measured at 12 h. All experiments were repeated at least three times with similar results using 3–5 mice per data point (*P<0.05, **P<0.01)

To investigate whether deletion of RIP3 is protective against autoimmune hepatitis, we challenged WT and RIP3^−/−^ mice with ConA. RIP3^−/−^ mice exhibited markedly reduced injury at 12 h (Figure 2a), had diminished elevations in serum levels of alanine transaminase (ALT; Figure 2b), and maintained relative normothermia (Figure 2c). Survival analysis suggested that RIP3^−/−^ mice exhibited delayed fatal injury, although differences in survival did not ultimately reach statistical significance (Figure 2d). Further, both serum (Figure 2e) and hepatic (Figure 2f) levels of pro-inflammatory cytokines were reduced in ConA-treated RIP3^−/−^ mice at 12 h, and there was a marked diminution in liver inflammatory infiltrate (Supplementary Figure S2A). Conversely, serum IL-10 – an anti-inflammatory cytokine – was elevated in RIP3^−/−^ mice compared with WT after ConA challenge (Figure 2g). There was no significant difference in the rates of TUNEL staining in ConA-treated RIP3^−/−^ *versus* WT liver (Supplementary Figure S2B). Notably, PBS-treated liver from WT and RIP3^−/−^ mice exhibited similar phenotypes (Supplementary Figure S3).

### RIP1 inhibition markedly exacerbates ConA hepatitis

**Figure 3:**
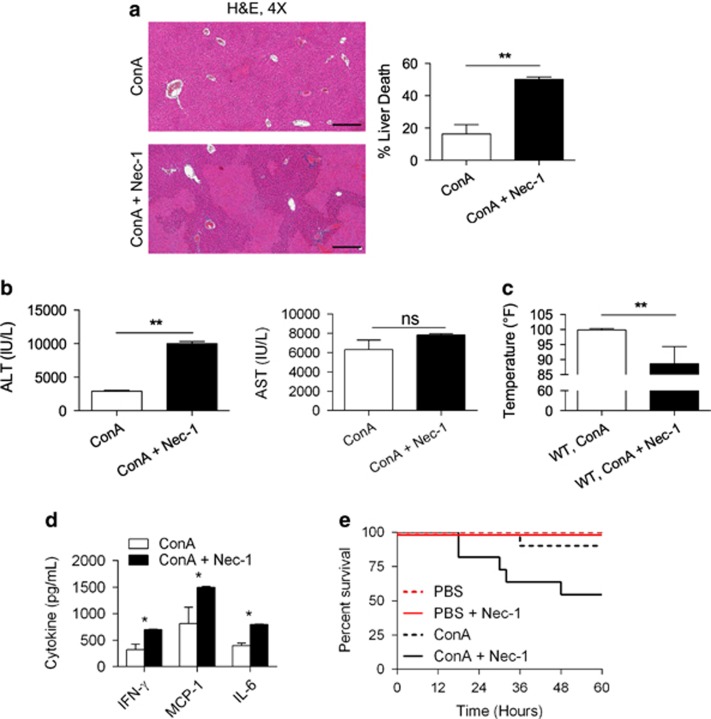
RIP1 inhibition exacerbates ConA hepatitis. (**a**) WT mice were treated with ConA (20 *μ*g/g) or ConA+Nec-1. Hematoxylin–eosin (H&E)-stained sections of the liver harvested 12 h after injury are shown, and the fraction of non-viable liver area was quantified (*n*=10/group) (scale bar=500 *μ*m). (**b**) Serum ALT, aspartate aminotransferase (AST), (**c**) change in core body temperature, and (**d**) serum cytokine levels were measured at 12 h (*P<0.05, **P<0.01). (**e**) Similarly, WT mice were treated with ConA (20 μg/g) or ConA+Nec-1 and observed for 5 days in a survival experiment (*n*=9/group; *P*=0.0001)

To determine whether RIP1 blockade is similarly protective against autoimmune hepatitis, we treated mice with Nec-1 prior to ConA administration. Nec-1 has been shown to inhibit necroptosis *in vivo* by blocking RIP1 activity.^26, 27, 28^ In contrast to our findings employing RIP3^−/−^ mice, RIP1 inhibition severely exacerbated disease. Specifically, ConA-challenged mice pretreated with Nec-1 exhibited exaggerated histological injury (Figure 3a) and elevated serum ALT compared with control mice (Figure 3b). The Nec-1-treated cohort also exhibited greater hypothermia (Figure 3c). Accordingly, serum levels of pro-inflammatory cytokines were higher after Nec-1 treatment in ConA-challenged mice (Figure 3d). Moreover, RIP1 blockade reduced animal viability after ConA challenge in survival experiments (Figure 3e). In the absence of ConA administration, Nec-1 had no appreciable effect on intrahepatic injury or systemic inflammation (Supplementary Figure S4). Importantly, RIP1 blockade using necrostatin-1s (Nec-1s) had similar effects on exacerbating liver injury and inflammation (Supplementary Figure S5).

### RIP1 inhibition in ConA hepatitis results in increased apoptotic cell death in hepatocytes

**Figure 4:**
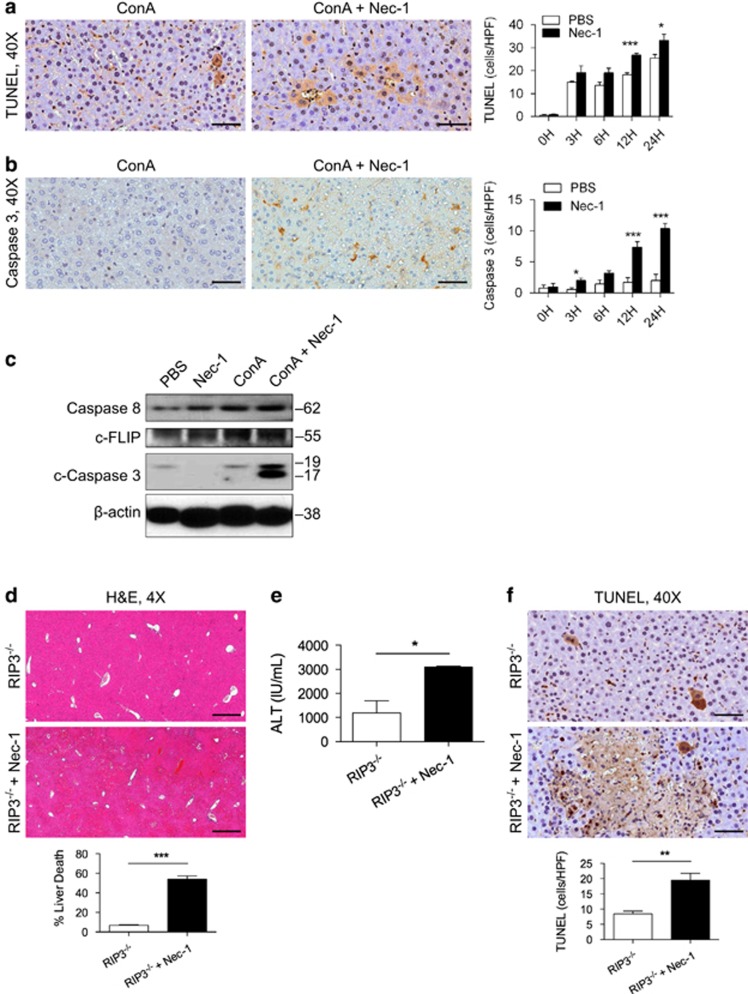
RIP1 inhibition diverts ConA-mediated hepatic insult to apoptotic injury. (**a**–**c**) WT mice were treated with ConA (20 μg/g) or ConA+Nec-1. (**a**) TUNEL (terminal deoxinucleotidyl transferase-mediated dUTP-fluorescein nick end labeling) staining and (**b**) cleaved Caspase 3 expression in hepatocytes were tested by immunohistochemistry at various time points and quantified (scale bar=50 *μ*m) (*n*=5/group). (**c**) Livers harvested at 12 h were tested for expression of Caspase 8, cleaved Caspase 3, and c-FLIP by western blotting. *β*-Actin was used as a loading control. (**d**–**f**) RIP3^−/−^ mice were treated with ConA (20 *μ*g/g) or ConA+Nec-1. (**d**) Hematoxylin–eosin (H&E)-stained sections of the liver harvested 12 h after injury are shown (scale bar=500 *μ*m), and the fraction of non-viable liver area was quantified. (**e**) Serum ALT was measured at 12 h, and (**f**) TUNEL staining was performed (scale bar=50 *μ*m) (*n*=7/group; *P<0.05, **P<0.01, ***P<0.001)

Notably, we found increased TUNEL staining (Figure 4a) and elevated expression of cleaved Caspase 3 (Figure 4b) in the Nec-1+ConA-treated liver compared with ConA treatment alone by immunohistochemistry. Similarly, on western blotting analysis full-length Caspase 8 and cleaved Caspase 3 were expressed at higher levels in the Nec-1+ConA group (Figure 4c). There was no difference in the expression of c-FLIP between the ConA and Nec-1+ConA groups (Figure 4c). Collectively, these data suggest that, in the context of ConA hepatitis liver injury, RIP1 inhibition increases apoptosis. Notably, administration of Nec-1 to ConA-challenged RIP3^−/−^ mice similarly exacerbated injury compared with treatment with ConA alone (Figures 4d and e). Likewise, Nec-1+ConA induced higher TUNEL staining in RIP3^−/−^ liver compared with treatment with ConA alone (Figure 4f).

**Figure 5:**
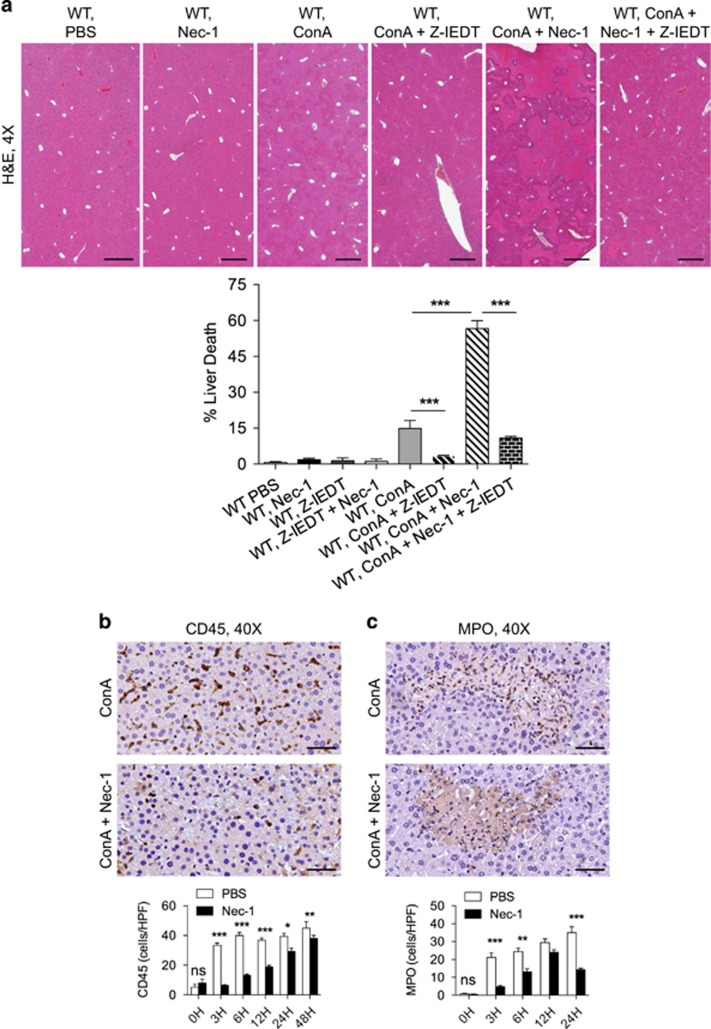
RIP1 inhibition in ConA hepatitis induces a non-inflammatory cell death, which is rescued by apoptosis blockade. (**a**) WT mice were treated with ConA alone (20 *μ*g/g), Nec-1 alone, Z-IETD alone, Nec-1+Z-IETD, ConA+Nec-1, ConA+Z-IETD, or ConA+Nec-1+Z-IETD (*n*=8/group). Hematoxylin–eosin (H&E)-stained sections of the liver harvested 12 h after treatment are shown, and the fraction of non-viable liver area was quantified (scale bar=500 *μ*m). (**b** and **c**) WT mice were treated with ConA (20 μg/g) or ConA+Nec-1 (*n*=8/group). (**b**) CD45^+^ pan-leukocytic infiltrate and (**c**) MPO^+^ neutrophilic infiltrate were tested at various time points. (scale bar=50 *μ*m; *P<0.05, **P<0.01, ***P<0.001)

To definitively test whether apoptotic cell death was a primary mechanism in the exacerbation of hepatocyte injury in ConA+Nec-1-treated animals, we blocked apoptosis using a Caspase 8 inhibitor. Caspase 8 blockade protected ConA+Nec-1-treated WT mice based on histological analysis (Figure 5a). Caspase 8 blockade similarly protected ConA- and ConA+Nec-1-treated RIP3^−/−^ mice (not shown). Consistent with apoptosis being classically considered a non-inflammatory form of cell death, we found markedly less immune infiltration of the liver in the ConA+Nec-1-treated liver compared with ConA alone, despite exacerbated hepatic injury. In particular, pan-leukocyte infiltration and intrahepatic neutrophilia were comparatively reduced in the ConA+Nec-1 liver (Figures 5b and c). These data suggest that blocking necroptosis via RIP1 inhibition in ConA hepatitis results in reduced intrahepatic inflammatory infiltrate associated with evidence of exacerbated apoptotic hepatocyte death.

### Blockade of RIP1 or RIP3 ameliorates APAP toxicity

**Figure 6:**
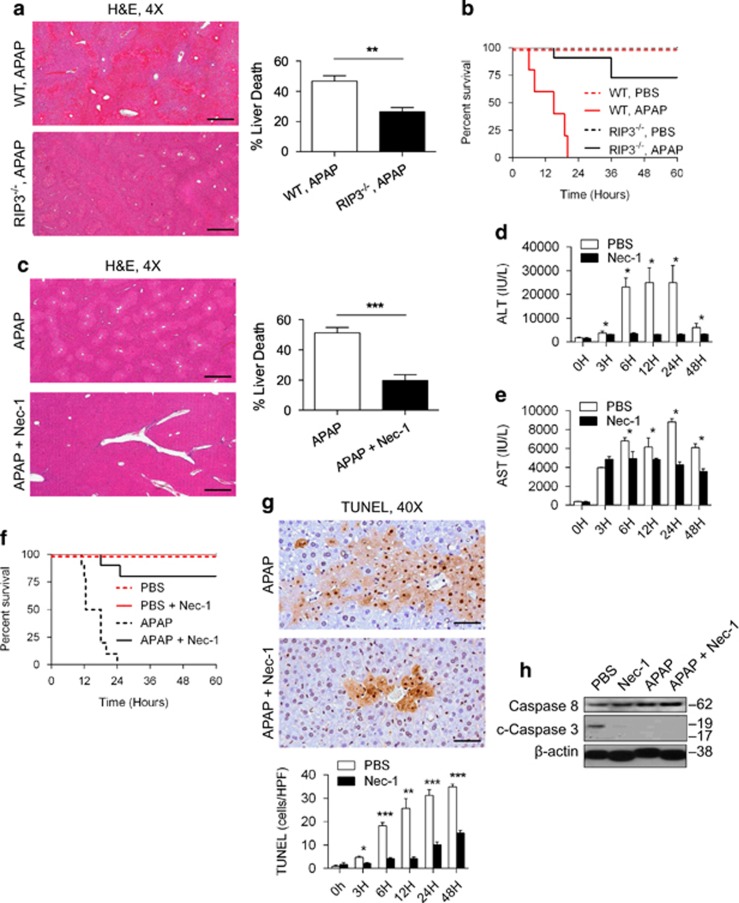
Blockade of RIP1 or RIP3 protects against APAP injury. (**a**) WT and RIP3^−/−^ mice were treated with APAP (500 *μ*g/g). Hematoxylin–eosin (H&E)-stained sections of the liver harvested 12 h after injury are shown, and the fraction of non-viable liver was quantified (*n*=10/group) (scale bar=500 *μ*m). (**b**) WT and RIP3^−/−^ mice were also administered a potentially lethal dose of APAP (700 *μ*g/g) and observed in a survival experiment (*n*=6–10/group; *P*=0.0001). (**c**–**e**) WT mice were treated with APAP (500 μg/g) or APAP+Nec-1 (**c**) H&E-stained sections of the liver harvested 12 h after injury are shown, and the fraction of non-viable liver area was quantified (*n*=10/group) (scale bar=500 *μ*m). (**d**) Serum levels of ALT and (**e**) aspartate aminotransferase (AST) were determined at serial time points after injury. (**f**) Similarly, WT mice were administered a potentially lethal dose of APAP (700 *μ*g/g) or APAP+Nec-1 and observed in a survival experiment (*n*=10/group; *P*<0.0001). (**g**) TUNEL (terminal deoxinucleotidyl transferase-mediated dUTP-fluorescein nick end labeling) staining was quantified in the APAP (500 *μ*g/g) and APAP+Nec-1 liver at various time points after injury (*P<0.05, **P<0.01, ***P<0.001). (**h**) Western blotting was performed for the indicated proteins using the livers of mice treated with PBS, Nec-1, APAP (500 *μ*g/g), or APAP +Nec-1

We next endeavored to determine whether RIP1 and RIP3 inhibition would similarly have opposite effects in an alternative model of acute liver injury. APAP-treated RIP3^−/−^ mice exhibited markedly diminished hepatocyte injury (Figure 6a) and prolonged survival (Figure 6b) compared with WT mice, as reported.^29^ However, in contrast to its effects on ConA treatment, RIP1 blockade also resulted in markedly diminished APAP injury based on analysis of liver histology (Figure 6c) and serum transaminases (Figures 6d and e). Further, Kaplan–Meier analysis revealed a marked survival advantage associated with Nec-1 treatment (Figure 6f). Nec-1s was similarly protective against APAP injury (Supplementary Figure S5). Notably, in contrast to the ConA model, Nec-1 treatment lowered TUNEL staining in the APAP-treated liver (Figure 6g). However, Nec-1 did very modestly increase full-length Caspase 8 expression in APAP-treated mice similar to our findings in ConA injury, whereas cleaved Caspase 3 was not upregulated (Figure 6h). Taken together, these data suggest that blockade of necroptosis via RIP1 inhibition has opposite effects on liver disease in APAP- and ConA-mediated injuries.

### RIP1 blockade in APAP-treated mice results in diminished inflammasome activation and reduced sterile inflammation

**Figure 7:**
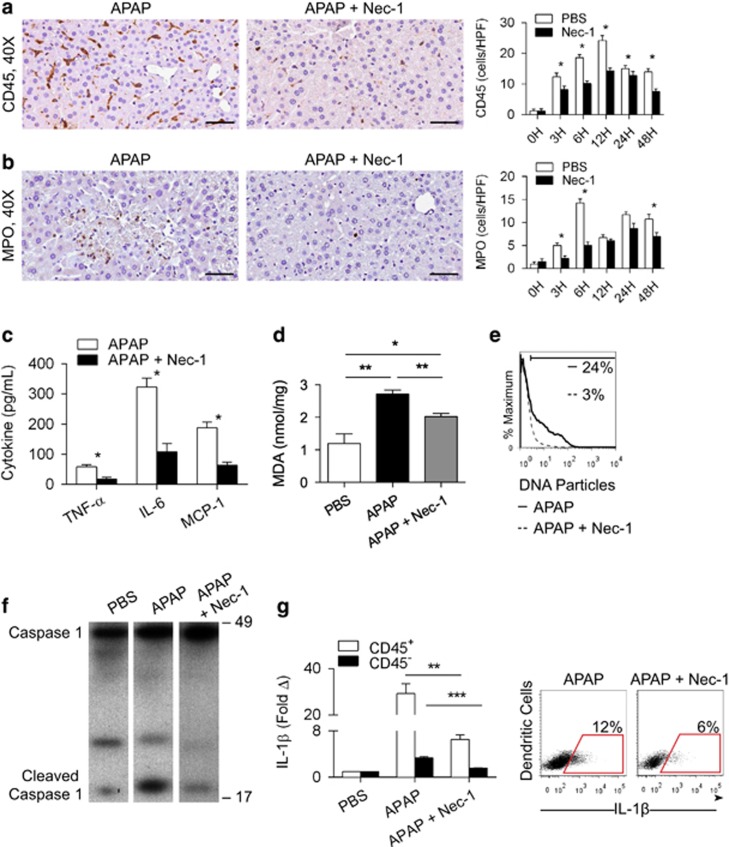
RIP1 blockade diminishes intrahepatic inflammation in APAP injury. WT mice were treated with APAP (500 μg/g) or APAP+Nec-1 (*n*=10/group). (**a**) CD45^+^ pan-leukocytic infiltrate and (**b**) MPO^+^ neutrophilic infiltrate were tested at various time points (scale bar=50 *μ*m). (**c**) At 12 h, serum levels of TNF-α, interleukin (IL)-6, and MCP-1 were measured. (**d**) Hepatic levels of malondialdehyde (MDA) at 12 h were determined for all the treatment groups. (**e**) Similarly, serum levels of DNA particles were determined by flow cytometry at 12 h in mice treated with APAP or APAP+Nec-1. (**f**) Treatment groups were tested for hepatic expression of Caspase 1 and cleaved Caspase 1 by western blotting. (**g**) IL-1*β* expression was tested at 12 h in CD45^−^ hepatic parenchymal cells, CD45^+^ hepatic leukocytes and by specifically gating on CD11c^+^MHCII^+^ dendritic cells. Experiments were repeated 2–4 times with similar results (*P<0.05, **P<0.01, ***P<0.001)

As APAP injury may be contingent on activation of the NLRP3 inflammasome,^17^ we postulated that the protection offered by inhibition of RIP1 was related to diminished inflammasome activation and consequently less robust intrahepatic inflammation. We confirmed that NLRP3^−/−^ mice are protected from APAP injury (Supplementary Figures S6A–D). Conversely, NLRP3^−/−^ animals were not protected against ConA hepatitis (Supplementary Figures S6E–G). In addition, neither ConA treatment nor ConA+Nec-1 induced IL-1*β* production in liver innate inflammatory cells (Supplementary Figure S6H). Further, consistent with our hypothesis, APAP-challenged WT mice treated with Nec-1 exhibited a diminished hepatic infiltrate of CD45^+^ leukocytes (Figure 7a) as well as markedly reduced neutrophilia (Figure 7b) at several time points compared with APAP treatment alone. Serum levels of TNF-α, IL-6, and MCP-1 were also reduced upon blocking necroptosis by inhibiting RIP1 in the context of APAP treatment (Figure 7c). Further, APAP+Nec-1-treated mice had reduced hepatic levels of ROS (Figure 7d) and serum DNA particles were less elevated (Figure 7e), both indicative of reduced sterile inflammation. To directly examine whether necroptosis inhibition is associated with reduced activation of the NLRP3 inflammasome, we tested whether APAP+Nec-1-treated WT mice had diminished Caspase 1 activation and IL-1*β* generation. As predicted, blocking RIP1 resulted in reduced hepatic levels of cleaved Caspase 1 after APAP treatment (Figure 7f). Further, both parenchymal and inflammatory cell expression of IL-1*β* were lower in the APAP+Nec-1 group (Figure 7g). Collectively, these data demonstrate that blocking RIP1 diminishes inflammasome activation, immune cell infiltration, and sterile inflammation after APAP administration. Similar evidence of suppressed inflammation (Supplementary Figure S7A) and diminished IL-1*β* expression was evident in APAP-treated RIP3^−/−^ mice (Supplementary Figures S7B and C).

## Discussion

Human autoimmune hepatitis can affect patients of all ages. Its definitive pathogenesis is uncertain, but it is widely believed that an environmental agent triggers an autoimmune process in genetically predisposed individuals.^5^ Environmental triggers include viruses, immunizations, herbs, and an array of medications and drugs.^30^ The primary genetic associations with autoimmune hepatitis pertain to the HLA region of the major histocompatibility complex (MHC) locus on chromosome six.^31^ The clinical manifestations of disease are variable and an individual's symptomatology can follow an undulating course. Diagnosis is based on clinical, histological, biochemical, and serological findings.^5^ Circulating self-reactive autoantibodies, elevated serum IgG, and an abnormal hepatic biochemical profile in the presence of histologically confirmed hepatitis support the diagnosis. Histological examination of the liver in patients with autoimmune hepatitis reveals a large cellular infiltrate in the portal tracts and adjacent parenchyma, including monocytes, macrophages, plasma cells, and T lymphocytes.^32^ Treatment with intensive immunosuppressive regimens usually induces disease resolution or remission; however, maintenance therapy is often required.^5^ In patients unresponsive to immunosuppressive therapy, liver transplantation may be necessary.^33^

ConA hepatitis is a well-described murine model of autoimmune hepatitis, but it differs from the human condition in its well-defined exogenous trigger and short injury course. Nevertheless, its MHC-related immune-mediated mechanism has facilitated understanding of human disease. ConA binds to MHC complexes on hepatic macrophages and Kupffer cells, modifying their MHC structure. CD4^+^ T cells immediately recognize the Con A-modified MHC on antigen-presenting cells and become activated, releasing IL-1 and IL-2. Conversely, CD8^+^ T-cell-mediated cytotoxicity has a relatively minor role in hepatic injury. The initial CD4^+^ T-cell injury and cytokine release results in a 'second hit' involving an array of innate inflammatory cells.^7, 8, 9^ In particular, NKT cells are critical in inducing hepatocyte injury in ConA-mediated hepatitis via a FasL-dependent mechanism.^34, 35^ Similarly, ligation of innate immune receptors – including TLR9 – by intrahepatic DAMPs generates cytokine release from macrophages and Kupffer cells, thus exacerbating injury.^36^

Regardless of immune-mediated mechanism, the ultimate result of autoimmune hepatitis in humans and in murine models is hepatocyte death. The goals of this study were to determine the respective roles of RIP1 and RIP3 in hepatocyte death in autoimmune hepatitis and to compare it with an alternate model of acute liver injury, specifically APAP. We show upregulated expression of RIP1 and RIP3 in both disease models. Further, RIP3 deletion was protective in both ConA hepatitis and APAP. However, whereas RIP1 inhibition was protective in APAP, it markedly exacerbated ConA hepatitis. This is evidenced by increased expression of cleaved caspase 3 in ConA+Nec-1-treated animals. RIP1 inhibition even exacerbated ConA-mediated injury in the context of RIP3 deletion. There are two novel findings that emerge from this: (i) RIP1 inhibition may have diametrically opposed effects in divergent models of acute liver injury, and (ii) blockade of different components of the necrosome may produce contrasting effects. Specifically, whereas RIP3 blockade protects against autoimmune hepatitis, RIP1 blockade markedly exacerbates disease, and blockade of either necrosome component is protective in APAP. These data imply that RIP1 and RIP3 cannot be simply considered part of a single functional unit.

A plausible explanation for our findings of the disparate effects of RIP1 inhibition in ConA hepatitis *versus* APAP injury may relate back to the immunological mechanism of injury. ConA is a primarily T-cell-mediated phenomenon in which we show that the NLRP3 inflammasome is completely dispensable and sterile inflammation has less of a central role.^7, 8, 9^ However, we found that in the APAP-injured liver the NLRP3 inflammasome is essential for disease progression. Hence, blocking necroptosis via inhibiting RIP1 or RIP3 eliminates a central mechanism of injury by reducing sterile inflammation.

It must be recognized that Nec-1 is known to inhibit IDO, which can have an important role in inflammatory disease settings.^26, 37^ However, it is unlikely that this substantially contributed to our results as we found a similar effect using Nec-1s, which has weaker IDO inhibition compared with Nec-1.^26^ Another limitation of our study is that RIP1 is not only a regulator of necroptosis but also an important regulator of immune responses and cytokine production, especially TNF-α signaling.^38^ Therefore, the systemic administration of Nec-1 can have collateral effects that are unrelated to hepatocyte necroptosis. Nevertheless, Nec-1 and Nec-1s remain standard modalities to block necroptosis via RIP1 inhibition. Importantly, our unusual dosing regimen of Nec-1 and Nec-1s intended to achieve effective RIP1 inhibition *in vivo* was probably excessive given its short half-life.^39^ We did, however, achieve similar findings using a single administration of Nec-1s immediately before ConA or APAP administration, suggesting that excessive dosing of inhibitors does not account for our findings (not shown).

Whereas necroptosis appears to be an active mechanism in ConA hepatitis as evidenced by markedly elevated RIP1 and RIP3 expression, apoptosis is also a parallel mode of hepatocellular death. This is supported by our observation of upregulation of Caspase 3 activity as well as our finding that blockade of apoptosis using Z-IETD is protective. Even more interestingly, we discovered that the mechanism for exacerbated injury in the context of RIP1 inhibition appears to be accentuation of apoptosis as evidenced by higher Caspase 3 activation in ConA+Nec-1-treated mice compared with animals treated with ConA alone. However, our findings must be appreciated in light of more recent data which suggest that RIP1 and RIP3 are not only involved in necroptosis induction but may also have a role in apoptosis. For instance, RIP1 can lead to apoptosis induction if RIP3 is inhibited by provoking a switch from necroptosis to apoptosis.^40, 41^

Our findings contrast somewhat with previous reports in the literature. Previous studies have shown that Nec-1 can protect against ConA hepatitis.^20, 25^ Further, other reports have shown that RIP3^−/−^ mice are not protected against ConA hepatitis.^42^ Indeed, much of the literature in acute liver injury is mired by conflicting reports between laboratories.^43^ For example, in APAP injury there are conflicting reports as to the pathogenic role of liver macrophages, neutrophils as well as the significance of the NLRP3 inflammasome.^17, 18, 44, 45, 46, 47, 48, 49^ The reasons for these inconsistencies are speculative. Variability between strains of mice and intestinal microbiomes between laboratories or even between treatment cohorts is a plausible explanation for these discrepancies. Additional confounding factors may be embedded within the precise experimental techniques used for studies. For example, use of exogenous DMSO alone as a solvent can induce substantial hepatic injury.^48^ Therefore, precise knowledge of the experimental parameters employed in murine models is necessary before translating discovery to human disease or experimental therapeutics.

## Materials and Methods

### Animals and hepatotoxicity models

Male C57BL/6 (H-2K^b^) mice (6–8-week old) were obtained from Jackson (Bar Harbor, ME, USA). RIP3^−/−^ mice were obtained from Genentech (San Francisco, CA, USA). NLRP3^−/−^ mice were a gift from Gabriel Núñez (University of Michigan, Ann Arbor, MI, USA). Littermates were used within each experiment at 8–10 weeks of life. To induce acute hepatic injury, mice were treated with ConA (20 *μ*g/g, IV) or APAP (500 *μ*g/g, i.p.; both Sigma-Aldrich, St. Louis, MO, USA). For survival experiments, we used higher doses of ConA (40 *μ*g/g, IV) and APAP (700 *μ*g/g). Changes in serum liver enzymes, including ALT and aspartate aminotransferase, were determined using commercial kits (Sigma-Aldrich). In selected experiments, mice were pretreated with Nec-1 (1.65 *μ*g/g, IP; Sigma-Aldrich) or Caspase 8 inhibitor (Z-IETD-FMK, 5 *μ*g/g, IP; BD, Franklin Lakes, NJ, USA) daily for 3 days prior to treatment. Alternatively, mice were pretreated with Nec-1s (BioVision, Milpitas, CA, USA) using a similar dosing regimen. Animal procedures were approved by the New York University School of Medicine IACUC.

### Cellular isolation

Liver leukocytes were isolated as previously described by us.^50^ Briefly, immediate postmortem laparotomy was performed, and the liver was infused with 0.1% Collagenase IV (Worthington Biochemical, Lakewood, NJ, USA). Hepatectomy was then performed and the livers were mechanically minced before incubation with Collagenase IV at 37 °C for 30 min. Low speed (50 × *g*) centrifugation was performed to exclude the pelleted hepatocytes followed by high speed (400 × *g*) centrifugation to isolate the hepatic non-parenchymal cells (NPC). The NPC were then further enriched over a 40% Optiprep (Sigma-Aldrich) gradient. In selected experiments, NPC were cultured at a density of 1 × 10^6^ cells/ml for 24 h before analysis of cell culture supernatant.

### Flow cytometry and cytokine analysis

Murine liver NPC were incubated with Fc blocking reagent (Biolegend, San Diego, CA, USA) for 15 min followed by incubation with fluorescent-conjugated mAbs directed against mouse CD45 (30-F11, no. 103134), CD11c (N418, no. 117318), or MHC II (M5/114.15.2, no. 107605; all Biolegend). For intracellular cytokine staining, freshly harvested liver NPC were incubated for 4–6 h with Brefeldin A (1 : 1000; Biolegend) before permeabilization of cells and staining using fluorescent-conjugated mAbs against IL-1*β* (3A6, no. 12242, Cell Signaling, Danvers, MA, USA). IL-10, IL-6, MCP-1, or TNF-*α* levels in serum or cell culture supernatant were determined using a cytometric bead array (BD). Malondialdehyde was measured using the Lipid Peroxidation Assay Kit (Abcam, Cambridge, MA, USA), and serum DNA particles was measured using propidium iodide (Biolegend).

### Histopathology and immunohistochemistry

For histological analysis, liver specimens were fixed with 10% buffered formalin, dehydrated in ethanol, and then embedded with paraffin and stained with hematoxylin–eosin. Percentage of cell death was determined as we have described.^10^ For immunohistochemical analysis, slides were stained for anti-mouse CD45 (no. 550539, BD), MPO (no. b6699, LS Biosciences, Seattle, WA, USA), Caspase 3 (no. 9664, Cell Signaling), RIP1 (no. bs-10055R-Cy3, Bioss Antibodies, Woburn, MA, USA), and RIP3 (no. AP7819b, Abgent, San Diego, CA, USA). TUNEL staining was performed using a kit (no. 17–141, Millipore, Billerica, MA, USA). Human liver specimens were stained using a mAb directed against RIP3 (no. AP7819b, Abgent). Light microscopic images were captured with a Zeiss Axioscope 40 microscope/camera system (Zeiss, Thornwood, NY, USA). Data were quantified by examining 10 high-powered fields per slide. The microscope objective used for histological images is indicated in each respective figure.

### Western blotting

For western blotting, total protein was isolated from 10 mg liver tissue by homogenization in RIPA buffer (50 mM pH7.4 Tris-Hcl, 150 mM NaCl, 0.5% Na-deoxycolate, 0.5% NP-40, 0.25% SDS, 5 mM EDTA) with Complete Protease Inhibitor Cocktail (Roche, Pleasanton, CA, USA). Proteins were separated from larger fragments by centrifugation at 14 000 × *g*. After determining total protein by Bradford protein assay, 10% polyacrylamide gels (NuPage, Invitrogen, Grand Island, NY, USA) were equiloaded, electrophoresed at 200 V, electrotransferred to PVDF membranes, and probed with monoclonal antibodies to *β*-actin (no. ab8227), c-FLIP (no. ab8421), FADD (no. ab24533), RIP3 (no. ab152130, all Abcam), RIP1 (no. 3493), Caspase 1 (no. 2225), Caspase 3 (no. 9664S), and Caspase 8 (no. 4790). Blots were developed by ECL (Thermo Scientific, Ashvile, NC, USA).

### Statistics

Data are presented as mean±S.E. Survival was measured according to Kaplan–Meier method. Statistical significance was determined by Student's *t*-test and log-rank test using GraphPad Prism 6 (GraphPad Software, La Jolla, CA, USA). *P*-values<0.05 were considered significant.

## Abbreviations

APAP: acetaminophen
ConA: Concanavalin-A
NAPQI: *N*-acetyl-p-benzo-quinoneimine
GSH: glutathione
ROS: reactive oxygen species
Nec-1: Necrostatin-1
Nec-1s: Necrostatin-1s
NPC: non-parenchymal cells
ALT: alanine transaminase
