# Supplementary figures and images for: Divergent effects of RIP1 or RIP3 blockade in murine models of acute liver injury
Source: Cell Death Dis. 2015 May 7;6(5):e1759–. doi: 10.1038/cddis.2015.126 (PMC4669705; doi:10.1038/cddis.2015.126)

Supplemental Figure 1

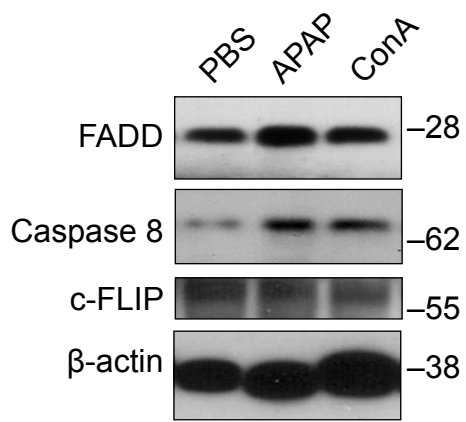

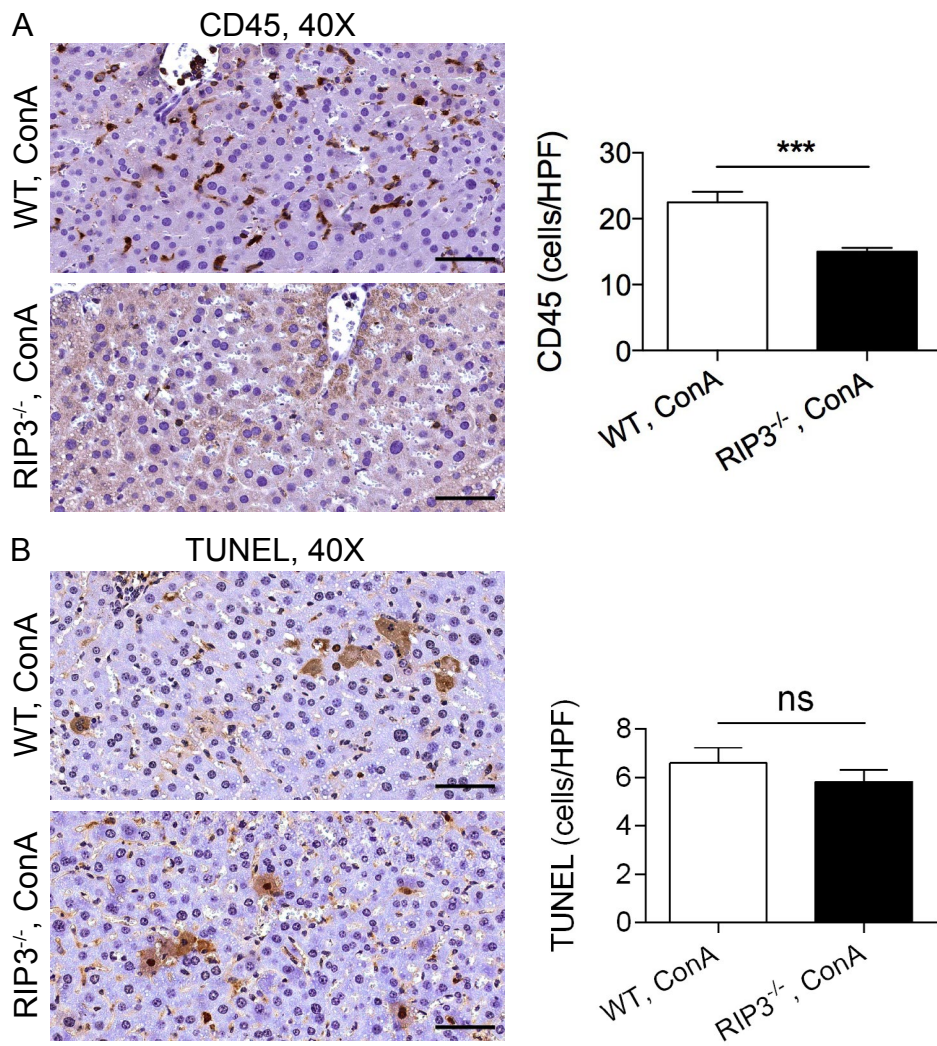

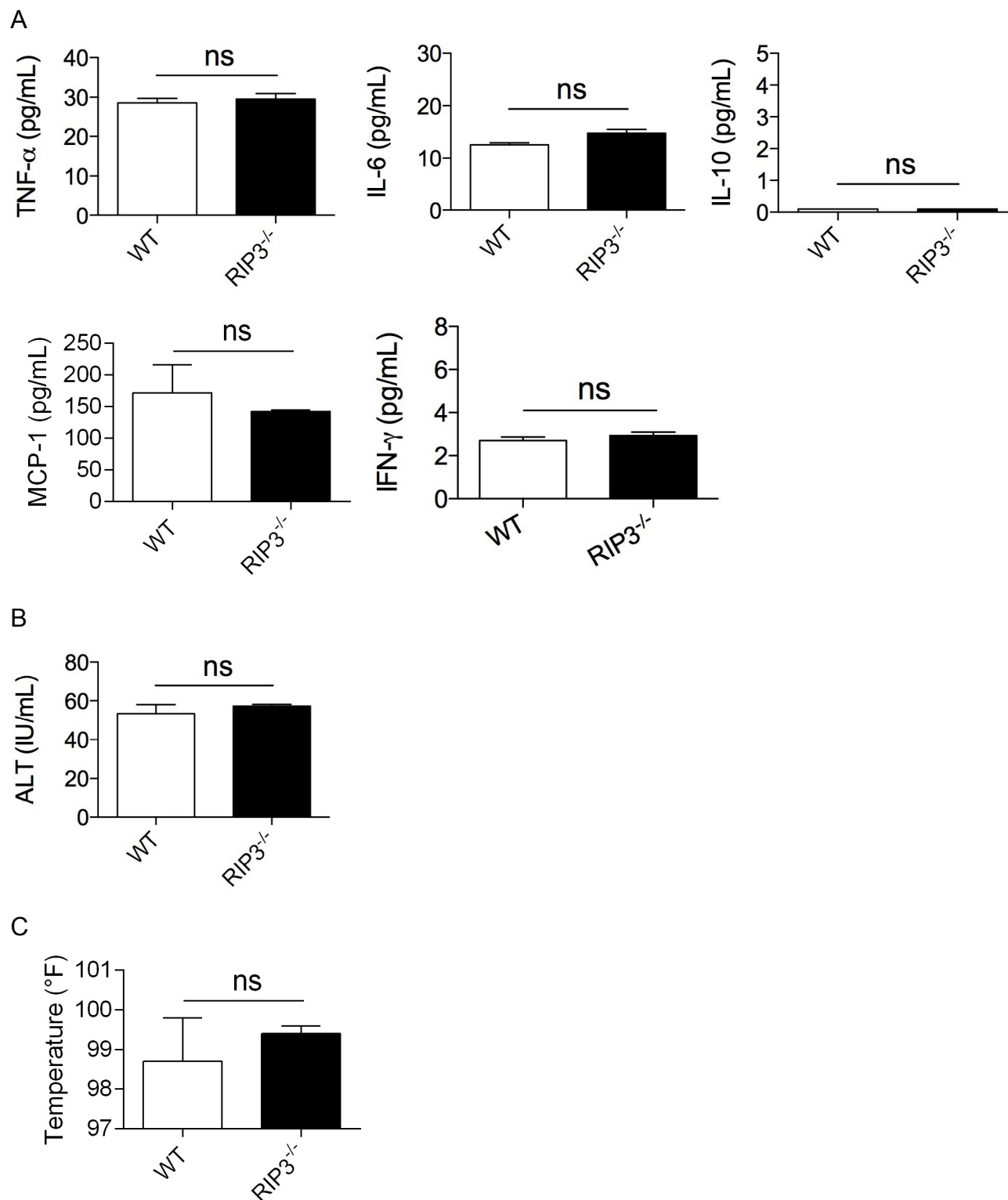

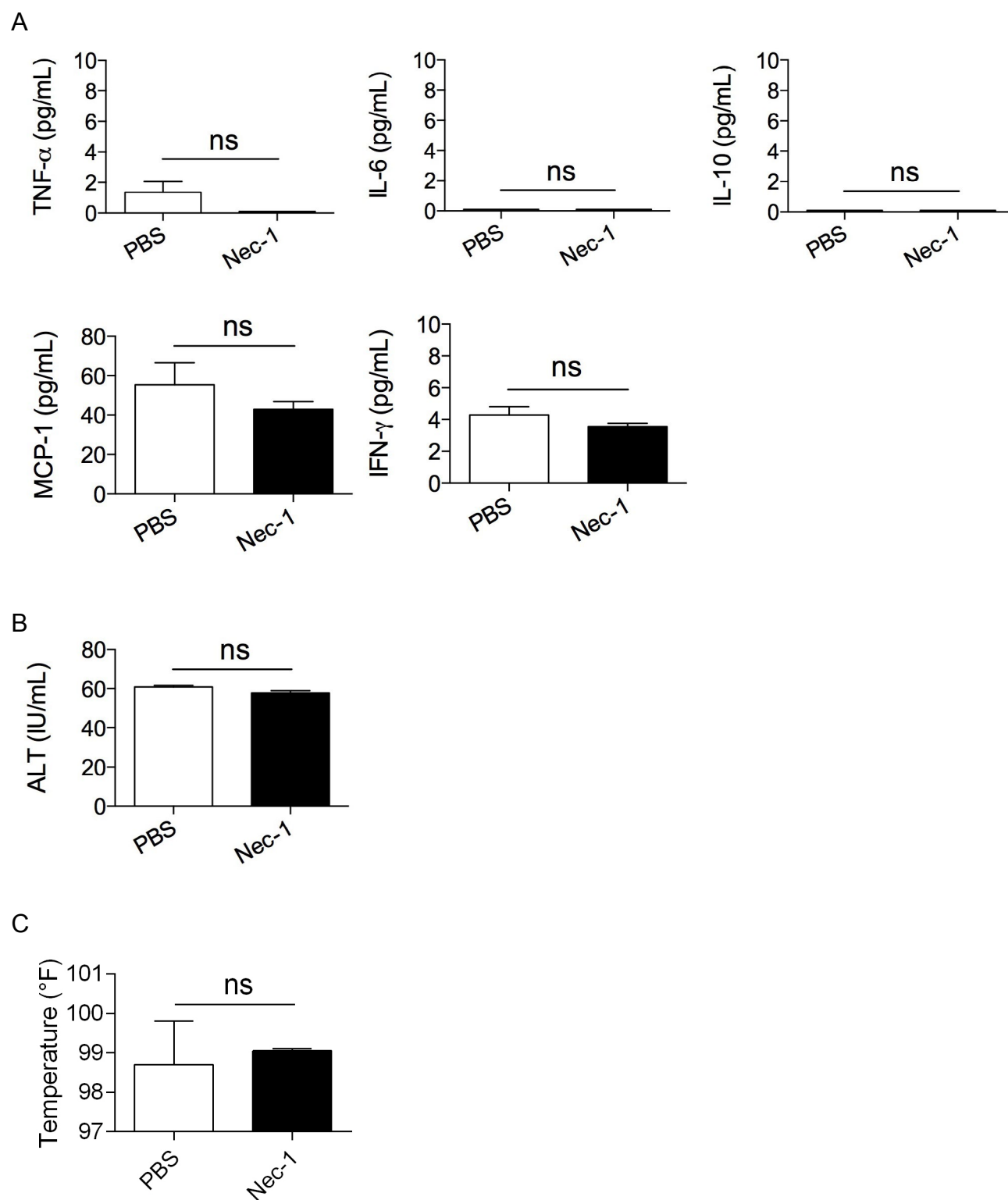

Supplemental Figure 5

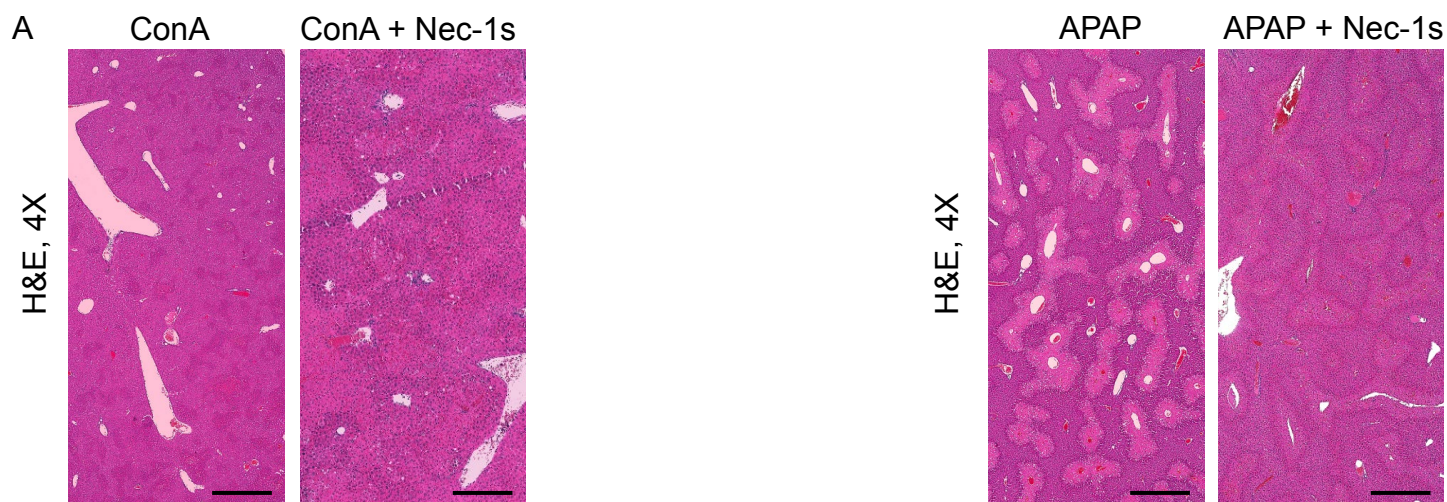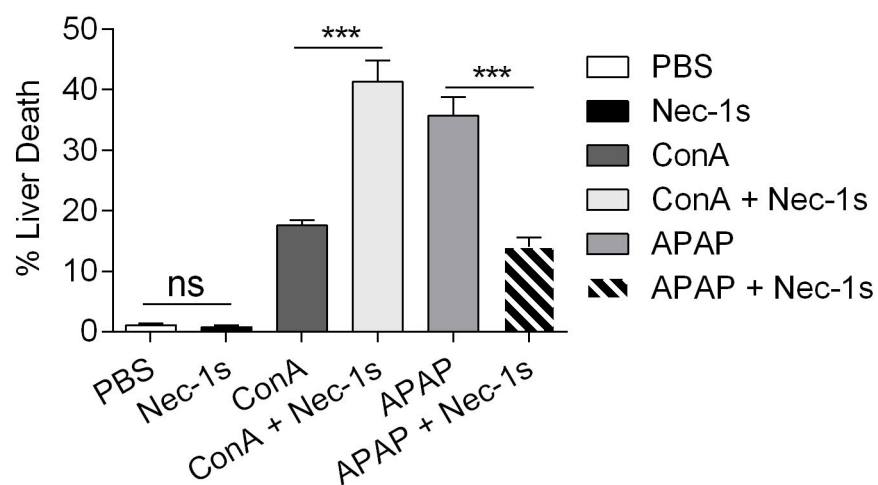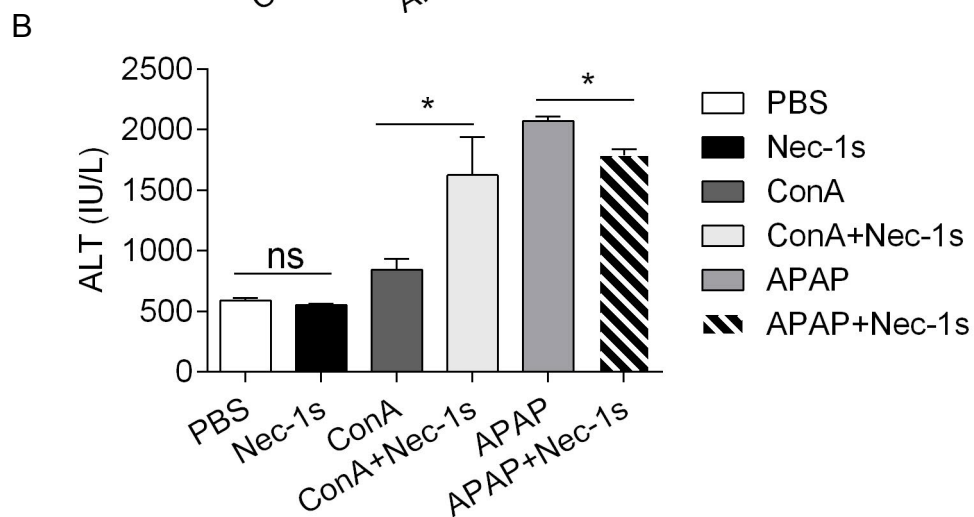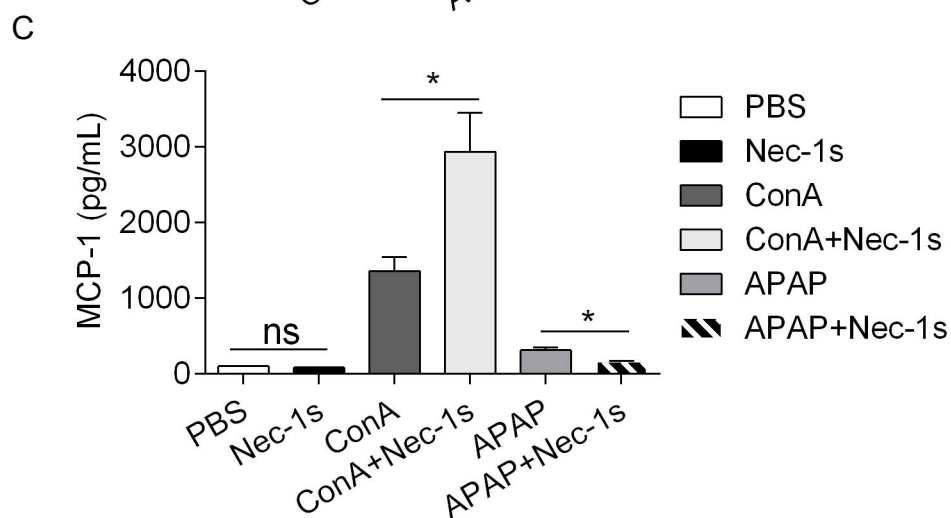

Supplemental Figure 6

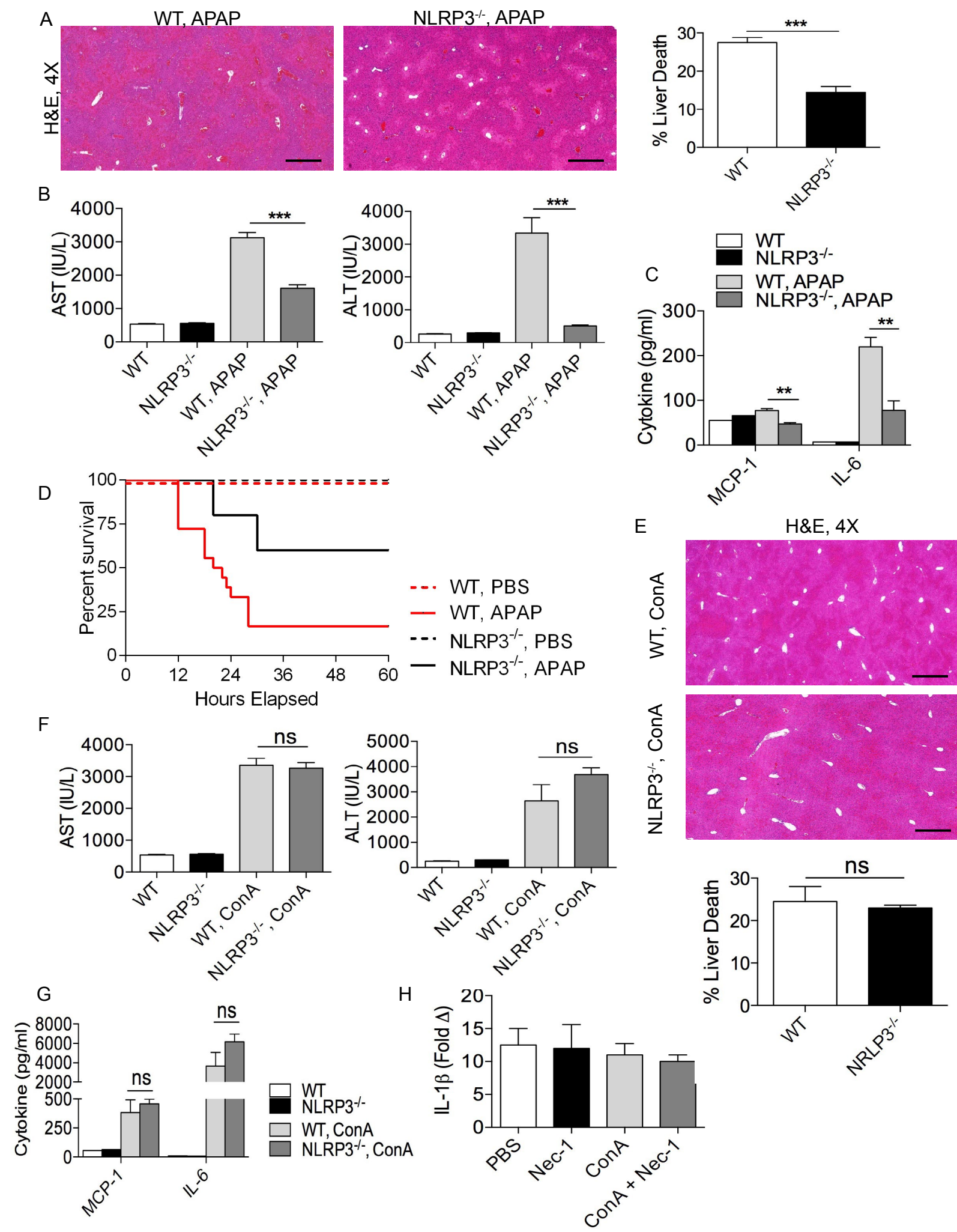

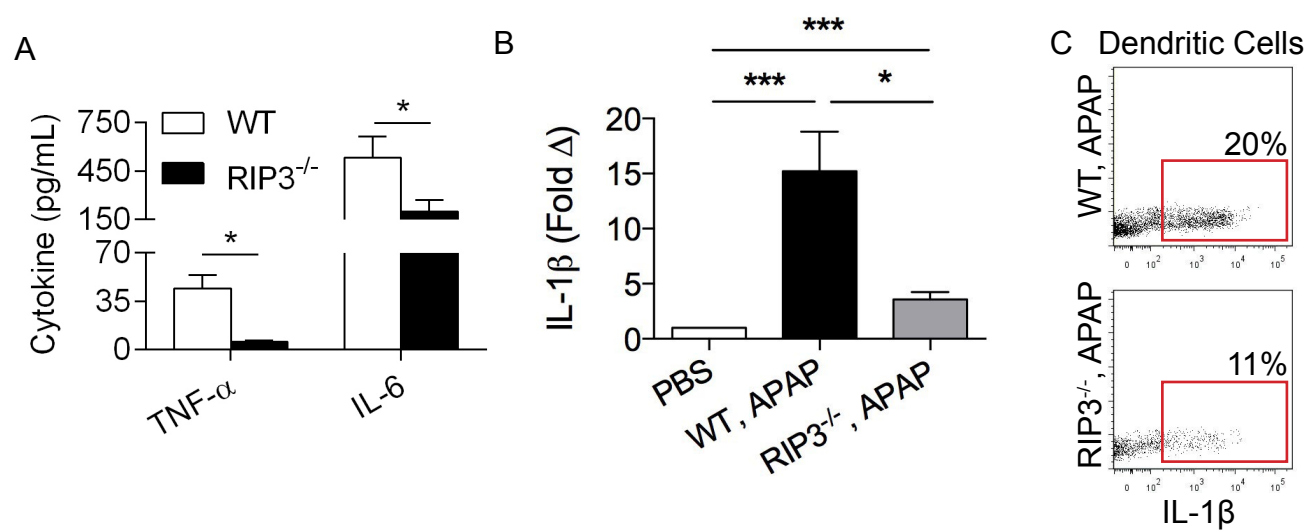

Supplement: Supplementary Figures [file cddis2015126x1.pdf]
