# Supplementary material for: Divergent effects of RIP1 or RIP3 blockade in murine models of acute liver injury
Source: Cell Death Dis. 2015 May 7;6(5):e1759–. doi: 10.1038/cddis.2015.126 (PMC4669705; doi:10.1038/cddis.2015.126)
Supplement: Supplementary Figure Legends [file cddis2015126x2.doc]

**Supplemental Figure Legends**

**Supplemental Figure 1. Upregulation of Caspase 8 and FADD in models of acute liver injury.** Mice were treated with PBS, APAP (500µg/g), or ConA (20μg/g) and tested for expression of FADD, Caspase 8, and c-FLIP at 12h by western blotting. β-actin was used as a loading control.

**Supplemental Figure 2. Deletion of RIP3 in ConA hepatitis results in decreased hepatic inflammatory cell infiltration but no change in apoptosis.** WT and RIP3^-/-^ mice were treated with ConA (20μg/g). **(A)** Hepatic infiltration with CD45^+^ inflammatory cells at 12h was quantified as was **(B)** TUNEL staining of liver (scale bar = 50µm; n=5/group; ***p<0.001).

**Supplemental Figure 3. RIP3^-/-^ and WT mice have similar characteristics at baseline.** PBS-treated WT and RIP3^-/-^ mice were tested for **(A)** serum levels of TNF-α, IL-6, IL-10, MCP-1, IFN-γ, **(B)** ALT, and **(C)** core body temperature (n=3/group). Data are representative of experiments repeated more than 3 times.

**Supplemental Figure 4. Mice do not exhibit inflammatory changes or hepatic injury in response to Nec-1 treatment.** PBS and Nec-1-treated WT mice were tested for serum levels of **(A)** TNF-α, IL-6, IL-10, MCP-1, IFN-γ, **(B)** ALT, and **(C)** core body temperature (n=3/group). Data are representative of experiments repeated more than 3 times.

**Supplemental Figure 5. Nec-1s exacerbates ConA hepatitis but protects against APAP injury. (A-C)** Mice were treated with PBS, Nec-1s, ConA (20μg/g), APAP (500µg/g), ConA+Nec-1s, or APAP+Nec-1s. (A) Livers were harvested at 12h and examined by H&E staining (scale bar = 500µm). The fraction of non-viable parenchyma was quantified. (B) Serum levels of ALT and (C) MCP-1 were measured (n=5/group; *p<0.05, ***p<0.001).

**Supplemental Figure 6. NLRP3^-/-^ mice are protected against APAP injury but not ConA hepatitis. (A-C)** WT and NLRP3^-/-^ mice were treated with PBS or APAP (500µg/g). **(**A) Livers were harvested at 12h and examined by H&E staining (scale bar = 500µm). The fraction of non-viable parenchyma was quantified. (B) Serum levels of AST, ALT, (C) MCP-1, and IL-6 were measured (n=5 mice/group; **p<0.01, ***p<0.001). **(D)** WT (n=18/group) and NLRP3^-/-^ (n=10/group) mice were treated with PBS or APAP (700µg/g). Survival was measured according to the Kaplan-Meier method (p<0.05). **(E-G)** WT and NLRP3^-/-^ mice were treated with PBS or ConA (20µg/g). (E) Livers were harvested at 12h and examined by H&E staining. The fraction of non-viable parenchyma was quantified. (F) Serum levels of AST, ALT, (G) MCP-1, and IL-6 were measured (n=5 mice/group). **(H)** WT mice were treated with Nec-1, ConA (20µg/g), or ConA+Nec-1 (n=3/group). Livers were harvested at 12h, dendritic cells were gated by flow cytometry and tested for expression of IL-1β compared with expression in animals treated with PBS (p=ns for all comparisons).

**Supplemental Figure 7. Decreased inflammasome activation in APAP-treated RIP3^-/-^ mice.** WT and RIP3^-/-^ mice were treated with APAP (500µg/g) and tested at 12h for **(A)** serum levels of TNF-α and IL-6. **(B)** CD45^-^ hepatic parenchymal cells were gated on flow cytometry and tested for IL-1β expression. **(C)** Similarly, CD11c^+^MHCII^+^ dendritic cells were gated and tested for expression of IL-1β. Experiments were repeated twice using 3 mice per group with similar results (*p<0.05, ***p<0.001).
